# Question Answering System Based on Knowledge Graph in Traditional Chinese Medicine Diagnosis and Treatment of Viral Hepatitis B

**DOI:** 10.1155/2022/7139904

**Published:** 2022-02-14

**Authors:** Yating Yin, Lei Zhang, Yiguo Wang, Mingqiang Wang, Qiming Zhang, Guo-zheng Li

**Affiliations:** ^1^Institute of Information on Traditional Chinese Medicine, China Academy of Chinese Medical Science, Beijing 100700, China; ^2^National Data Center of Traditional Chinese Medicine, China Academy of Chinese Medical, China; ^3^Experimental Research Center, China Academy of Chinese Medical Science, Beijing 100700, China; ^4^Information Office, Henan University of Chinese Medicine, Zhengzhou 450046, China

## Abstract

This article uses the real medical records and web pages of Chinese medicine diagnosis and treatment of hepatitis B to extract structured medical knowledge, and obtains a total of 8,563 entities, 96,896 relationships, 32 entity types, and 40 relationship types. The structured data was stored in the Neo4j graph structure database, and a knowledge graph of Chinese medical diagnosis and treatment of hepatitis B was constructed. The knowledge map is used as a structured data source to provide high-quality knowledge information for the medical question and answer system based on hepatitis B disease. Applying the deep learning method to the question identification and knowledge response of the question answering system makes the hepatitis B medical intelligent question answering system has important research and application significance. The question-and-answer system takes aim at hepatitis B, a public health problem in the world and leverages the advantages of traditional Chinese medicine for diagnosis and treatment. It provides a reference for doctors' disease diagnosis, treatment, and patient self-care. Its value is important for the treatment of hepatitis B disease.

## 1. Introduction

Traditional Chinese medicine, as China's traditional medicine, is a medical science oriented towards patient experience, which is mainly based on clinical practice and clinical trials [1]. Different from Western medicine, TCM medical records are often directly involved in the evaluation of curative effects of a given drug. It records the symptoms, syndrome types, prescriptions, and traditional Chinese medicines in detail [2]. The structured extraction and storage of clinical notes, the use of the internal correlation of mining data, and the extraction of practical and repeatable clinical strategies can be better referenced and used.

Hepatitis B virus (HBV) has become a worldwide public health problem since it was discovered in the sera of Australian aborigines in the 1960s. Hepatitis B is widespread in countries around the world, and a small number of patients can transform into cirrhosis or liver cancer. It is estimated that about 2 billion people in the world have been infected with hepatitis B, and about 350 million people have chronic hepatitis, of which 15%-20% can develop into cirrhosis and liver cancer, and the number of deaths per year is 500,000-750,000 [3]. At present, Western medicine adopts symptomatic treatment strategies including antivirus, protecting liver and lowering enzymes, reducing yellowing, and improving immunity [4]. Among them, antivirals often use entecavir, adefovir dipivoxil, etc. However, long-term use can cause drug resistance and adverse reactions to the kidneys [5]. In the long-term development of traditional Chinese medicine, it has accumulated rich experience in the prevention and treatment of hepatitis B, and many empirical prescriptions have emerged, such as Yinchenhao Decoction, Sini San, and Ganlu Xiaodu Dan, which are favored for their remarkable curative effect [6]. The multicomponent synergistic effects of traditional Chinese medicine compound prescriptions for treating hepatitis B, simultaneous targeting of multiple targets and multiple pathways, are more likely to be widely recognized by clinicians and patients and are more meaningful to medical researchers.

**Figure 1:**
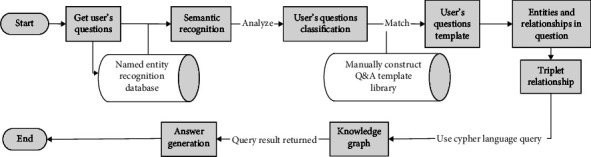
Work flow chart of hepatitis B intelligent question answering system.

Question answering system is an important research direction of natural language processing in the field of artificial intelligence. Compared with traditional search engines, Q&A systems can obtain knowledge and information conveniently, quickly, and efficiently [7]. However, in traditional question answering systems that use unstructured data as the source of knowledge, the convenience and timeliness of retrieval have been reduced. The knowledge graph technology provides a structured and real-time means of extracting a large amount of documents and is a structured and related data source [8]. This paper studies the structured knowledge graph as the data source of the question answering system, with the aid of deep learning methods, which not only saves the time of scree information but also provides high-quality data information. In addition, our data source combines real evidence and encyclopedic knowledge, which conforms to the actual occurrence of the disease, and also combines professional knowledge. The specific workflow of the research system is shown in Figure 1.

## 2. Related Work

The knowledge graph was first developed from in the context of semantic networks. Since it was proposed by Google in 2012, it has gained wide attention and application [9]. In essence, the knowledge graph is a knowledge base covering graph structure, which enables the knowledge graph to effectively store the association relation between data and knowledge [10]. With the rapid development of natural language processing in artificial intelligence, question and answering system, which was proposed in the 1960s when artificial intelligence research started, gradually entered the intelligent stage [11]. In recent years, the combination of knowledge graph and intelligent Q&S is a hot topic, but the combination and application of the two are rare in the field of medical textual [12].

Due to the variety of medical textual data sources, the Q&A structures are just as diverse. Many researchers have done some work for it. For instance, Cao et al. [13] designed an online Q&A system called AskHermes, which used health-related knowledges to help patients stay healthy. Other author has done similar studies; Sharma et al. [14] designed the Health On the Net Foundation (HONQA) for health related. But Sharma's system used two languages: English and French. Cairns et al. [15] used a large-scale document collection to leaen to answer real-world medical questions.

Compared with the above studies using popular medical science information on the web, some of the following studies are targeted at medical specialties. Cao et al. [16] designed a question answering system for primary liver cancer based on the knowledge graph, which can effectively answer questions about drugs, diseases, and symptoms related to primary liver cancer. Ma [17] designed thyroid disease Q&A; the system can effectively answer questions about the patient's thyroid diseases such as medications and examinations and save doctors' time, and doctors can use this system to make relevant queries on patients and prescriptions, which is more rapid and convenient. This paper is one of these studies that focus on specialist disease.

## 3. Materials and Methods

### 3.1. Materials and Data Standardization

#### 3.1.1. Medical Record Data

**Figure 2:**
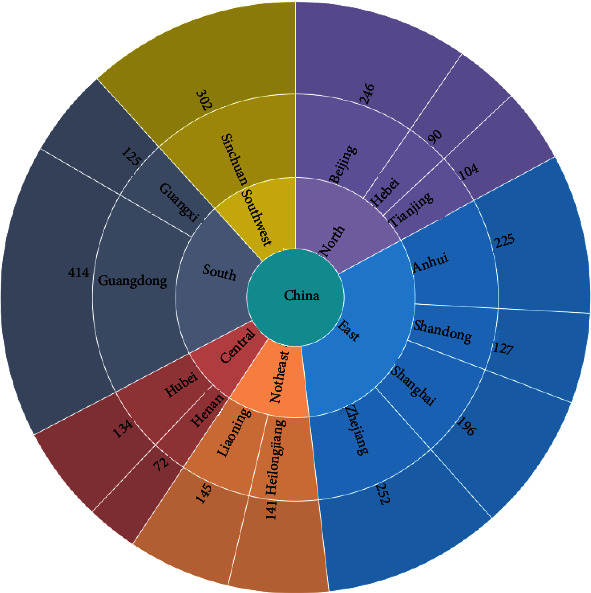
Source distribution map of medical records (seven-area method).

In this paper, we collected the electronic medical record information for viral hepatitis B from 8,544 patients treated by 117 doctors in 20 Grade A hospitals in different regions of China from 2009 to 2011.The data included 25,549 clinical records. Two TCM research were engaged entire time in filter data. The standard of data filtering is the lack of complete medical information of patients in medical records, such as pulse condition, tongue condition, TCM diagnosis, and TCM prescription will be deleted. Finally, the manuscript uses the medical records of 2573 first-time patients. The entire treatment process of these patients used only traditional Chinese medicine methods. Each record contains the time of visit, the age, sex, occupation, symptom description, medical history, anamnesis, diagnosis, TCM differentiation, prescription, and laboratory indicators. The detailed data information is shown in Figure 2.

#### 3.1.2. Medical Knowledge Base Data

**Figure 3:**
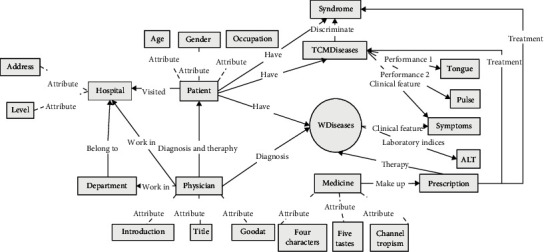
Ontology layer construction of medical record extraction.

**Figure 4:**
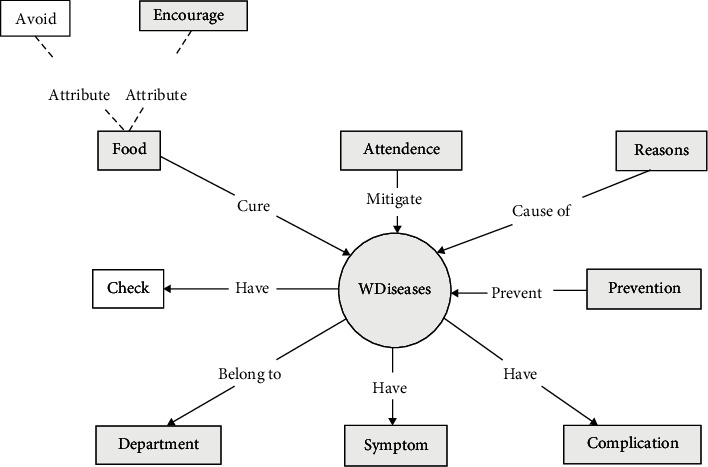
Ontology layer construction of supplementary data on the medical website.

The supplementary medical knowledge of hepatitis B in this study came from Xunyiwenyao, a vertical medical website (https://www.xywy.com/). The crawler script was used to crawl structured data and construct a medical knowledge map centered on hepatitis B. Traditional Chinese medicine focuses on self-regulation, so this knowledge graph focuses on daily self-recuperation, including recuperation diet, suitable food, and taboo food. Combining the above two approaches, we obtain the entity and relationship triad associated with hepatitis B. Detailed statistics on entities and relationships are shown in Table 1. The specific entity categories and relationship categories of the medical record knowledge graph are shown in Figure 3. The entity types and relationships of the hepatitis B nursing knowledge graph crawled on the Xunyiwenyao website can be seen in Figure 4.

#### 3.1.3. Data Standardization

For the supplementary medical knowledge from the Xunyiwenyao web, it had been finished data standardization. In medical record data, TCM symptom terms in extracted entities are classified and normalized by referring to “Diagnostics of TCM” [18] and “Differential Diagnostics of TCM Symptoms” [19]. According to “The Traditional Chinese Medicine Pharmacopoeia” [20], the alias and processing methods of traditional Chinese medicine are standardized. The diagnosis of hepatitis B disease by western medicine is based on the “Consensus on the Treatment of Chronic Hepatitis B in the Asia-Pacific Region” [21]. The diagnosis of traditional Chinese medicine is based on the standards indicated in the “Guidelines for Diagnosis and Treatment of Chronic Hepatitis B in Traditional Chinese Medicine” [22] to standardize terms such as diseases and symptoms of traditional Chinese medicine.

### 3.2. Knowledge Graph Methods

#### 3.2.1. Knowledge Acquisition

For medical record text data, first we designed the ontology layer of knowledge graph according to the overall structure of medical record, as shown in Figure 3. A joint model leveraging a multihead mechanism was used to extract entities and relationships [23]. Considering the limited accuracy of the model, the extraction results were proofread by a doctor of traditional Chinese medicine and two attending doctors of traditional Chinese medicine.

Based on the entity and relationship structure designed in Figure 4. For supplementary data on the medical website, Python scripts are used to perform xpath parsing of structured data on the web page. Crawler module uses urlib library, and data is temporarily stored in MongoDB database.

#### 3.2.2. Knowledge Representation

The triplet is a general representation of the knowledge graph, that is, **G** = (**E**, **R**, **S**), where **E** = {**e**_1_, **e**_2_, ⋯, **e**_∣**E**∣_} is the set of entities in the knowledge base, which contains a total of ∣**E**∣ kinds of different entities. **R** = {**r**_1_, **r**_2_, ⋯, **r**_∣**E**∣_} is the set of relations in the knowledge base, including ∣**R**∣ different relations. **S**⊆**E** × **R** × **E** represents the set of triples in the knowledge base. The basic form of triples mainly includes entity 1, relationship, entity 2 and concepts, attributes, attribute values, etc. [24]. In this study, taking TCM diseases and tongue diagnosis as an example (TCMdiseases, Performance1, Tongue) represents entity and relationship design; taking hospital and address as an example (Hospital, Address) represents attribute and attribute value design.

#### 3.2.3. Knowledge Storage

The final data storage of this study is a graph database. The storage principle of graph database is to use the nodes, edges, and attributes of graph to store graph data. We used the currently popular open source graph database Neo4j to store knowledge graphs. Neo4j provides Cypher language to import data and query graph data. Cypher is a descriptive graph query language with simple syntax and supports various graph mining algorithms [25].

### 3.3. Question and Answering Methods

#### 3.3.1. Medical Entity Recognition

In terms of named entity recognition, compared with English, Chinese requires word segmentation and is more difficult. In addition, the entity naming rules in the medical field are complex. In order to solve the above problems, Huang et al. [26] used Bi-LSTM (bi-directional LSTM) + CRF model. Its working principle is to implement named entity extraction with small granularity. The extracted results include a part of continuous entities and noncontinuous entities. On this basis, postprocessing is done on the extraction results to assemble the correct complex entities. Since postprocessing relies on a large number of manual construction rules, Li et al. [27] use deep learning to construct a classifier to complete postprocessing, that is, use Bi-LSTM to construct a dependency analysis tree, and use the shortest dependency path to determine whether to combine the entities extracted in the first step into a new entity, which replaced the original manual design rule and achieved the same effect as the manual construction rule in the experimental results. This article uses the Bi-LSTM+CRF model based on deep learning to achieve named entity recognition, which is precision is 0.8234, recall is 0.7241, and an F1-increased is 0.771.

#### 3.3.2. Question Template Matching

In the current research, three methods based on template matching [28], retrieval model, and deep learning [29] are applied in the field of problem understanding. The question answering system of this study currently only uses template matching. We design the question template according to the common questions of patients and use the Word2Vec lexical vector method [30] to calculate the text similarity between the question and the design template to complete the matching. Partial matching templates are shown in Table 2. This method reduces a lot of manual labeling and training corpus deep learning time, but in the follow-up, we will try to integrate deep learning technology into our system to make the system more intelligent.

#### 3.3.3. Knowledge Graph Query

When the user enters the inquiry request of hepatitis B disease, the intelligent question answering system for hepatitis B traditional Chinese medicine diagnosis and treatment first performs semantic analysis and word segmentation on the input text, performs matching and entity recognition extraction with the designed template library, and queries the relationship and relationship in the knowledge graph through the question template structure mapping of names and medical entity names; Neo4j's built-in Cypher language generates query statements based on rules [25]; the results of returned graph queries are output in natural language.

## 4. Experiment

All of our work were trained on the environment with Inter Core I5-4210M 2.60 GHz, with 4 of RAM, and with Windows 7 flagship operating system. Python programming language was used to write entities and relationships into Neo4j graph database for storage and display. Python works in an environment of PyCharm 2017.3.3, which was used to web crawlers and builded Q&S.

This paper's research steps can be summarized as follows:

1. When the user enters a question into the system, the system will use the deep learning model and the named entity recognition database established in the background to perform entity recognition and intent recognition for the sentence
2. After identifying the entities in the sentence, classify the entities involved. Match the artificially constructed question and answer template to predict the intention template in the user's sentence
3. The system matches and calculates to form a new question template and builds a triple structure that can be queried in the knowledge graph with the existing entities and relationships in the new sentence
4. Use Cypher language to query the answer in the knowledge graph and return the output

## 5. Results

### 5.1. Knowledge Graph

Based on real-world doctors' medical records and hepatitis B supplemental care data crawled from web pages, the constructed knowledge map is partially displayed as shown in Figure 4.

**Figure 5:**
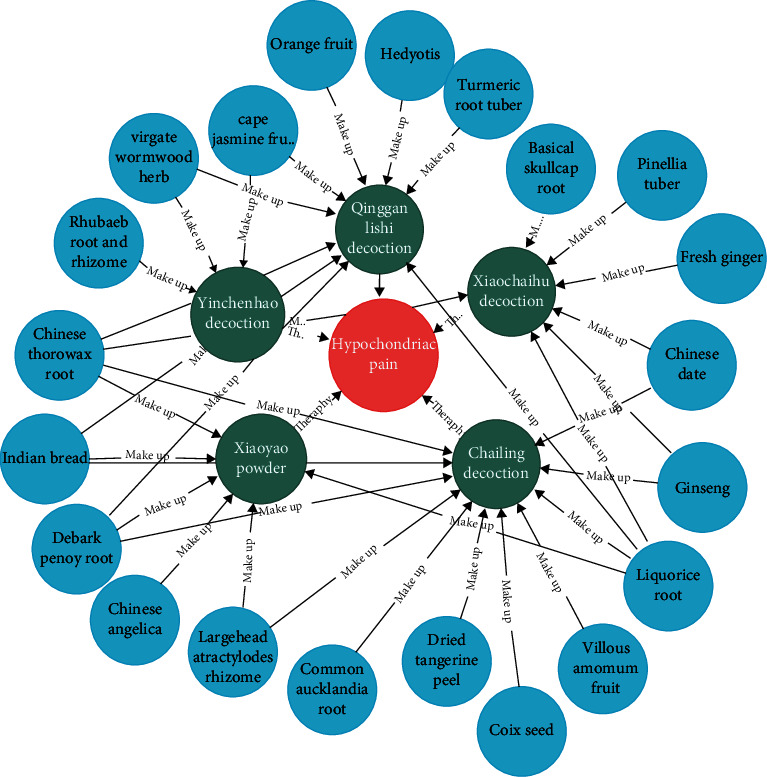
Partial display of hepatitis B knowledge graph (example: hypochondriac pain).

Figure 4 shows the knowledge map of the two-level relationship between Chinese medicine disease entities and prescription entities and the composition of Chinese medicines. As far as the hypochondriac pain of TCM disease is concerned, the commonly used prescriptions in TCM treatment plan are Yinchenhao decoction, Xiaoyao powder, Qinggan Lishi decoction, Xiaochaihu decoction, and Chailing decoction. As can be seen from Figure 5, for example, Xiaoyao powder is composed of six Chinese medicines: liquorice root, Chinese angelica, Indian bread, debark peony root, largehead atractylodes rhizome, and Chinese thorowax root.

### 5.2. Question and Answering System

We manually designed 300 TCM diagnosis and treatment hepatitis B-related questions with similar semantics to the template question and evaluated the answer output by the question and answer system against the original medical record and website crawling data to verify the performance of the question and answer system designed in this study. The system can recognize that the named entity is accurate, the question template matches the semantics, and the feedback output after the knowledge graph query is valid; then, the question is determined to be answered correctly. It can be seen from the experimental results that 81.67% of artificially designed questions can be answered correctly.

**Figure 6:**
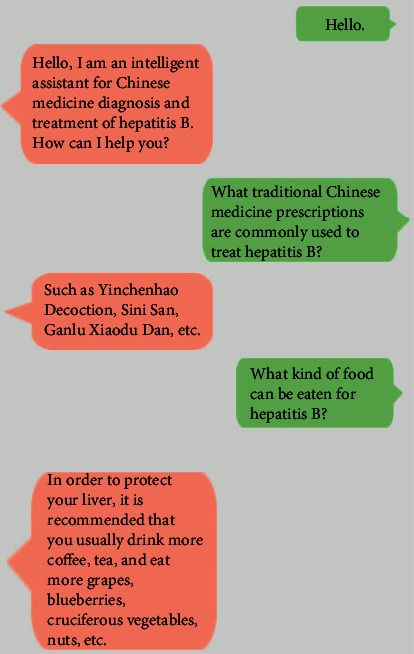
Partial display of Q&A system.

When users (including doctors, patients, and those in need) use our system, the designed hepatitis B intelligent diagnosis and treatment assistant will communicate with users. The assistant will answer the user's questions related to hepatitis B treatment, such as introduction to hepatitis B, traditional Chinese medicine prescriptions for hepatitis B, and dietary treatment methods for hepatitis B. Examples are shown in Figure 6.

In Figure 6, for the first step, the user first greets the assistant; the assistant introduces his identity and says hello to the user, asks what can help the user, and guides the user to ask questions. For step 2, when the user asks about the traditional Chinese medicine prescriptions that are commonly used to treat hepatitis B, the assistant will feed back to the user based on the search results of the background knowledge map. The commonly used prescriptions are Yinchen Hao decoction and Sini San. The user can add or subtract traditional Chinese medicine to the recommended Chinese medicine prescriptions according to the condition of the disease or after further confirming their needs. For step 3, the user asks about food recommendations that are beneficial to hepatitis B in daily life, and the assistant will calculate the recommendation to drink more tea and coffee and eat more grapes, blueberries, etc. Users can adjust hepatitis B by eating more food recommended by the assistant according to their preferences.

## 6. Discussions

Question answering systems have been proposed since the beginning of the development of artificial intelligence and have been applied in many fields since then. However, there are few developments in the medical field, especially those that use real-world medical records as data sources. The reason is, on the one hand, the complexity of medical knowledge itself, and there is no fixed pattern for the relationship between various entities. On the other hand, the medical record contains the patient's privacy, and it is difficult to obtain valuable data for research. At the beginning of the research of this paper, we started from the needs of users. First, we designed the ontology layer structure of the knowledge graph and then extracted the data according to the ontology layer. The experimental results show that the knowledge graph is a good form of knowledge organization. In the later stage, based on the knowledge map, the question and answer system is designed to realize the output of knowledge, which is more suitable for clinical use habits. The research ideas in this article can provide research ideas for the intelligent inheritance and application of doctors' medical records.

The amount of data collected at the beginning of this article is relatively large, but after filtering, the amount of data is relatively small. It can be seen that if you want to build a sufficiently intelligent and comprehensive medical question and answer system, not only must there be enough data sources, but the quality of the data cannot be ignored. The limitation of the question answering system constructed in this research lies in the intent analysis of the question. We use the template matching method. Using this method, once the problem is not in the template we manually designed, the content of the system feedback is not related to the problem. The next step is how to build a deep learning model to improve the accuracy of problem understanding and automatically expand the problem database. It will not only reduce a lot of work for researchers but also enhance the intelligent processing of the system, so as to improve retrieval efficiency and practicability.

## 7. Conclusion

As the basic form of recording patient visits, TCM clinical medical records record a large number of experiences that can reflect disease characteristics and diagnosis and treatment, and have important clinical guiding significance. As a major infectious disease in the world, hepatitis B not only takes away the happiness of people's lives but also threatens their lives. Traditional Chinese medicine is a treasure in Chinese traditional culture. It has a history of 5,000 years. It is famous for its holistic nursing concept, the synergistic effects of multiflavored Chinese medicine, and its low side effects. Treating hepatitis B with traditional Chinese medicine can become a reliable treatment for the disease.

In this paper, we build a hepatitis B intelligence question and answer system based on the hepatitis B knowledge graph, starting from solving the actual needs of patients and applying deep learning technology to the medical field. It provides a research idea opportunity for applying artificial intelligence technology to medical records in the real world in the future. Using knowledge graphs to store and display data can more intuitively and quickly dig out the laws of data connotation and apply them. Of course, doctors are also a great profession. As far as current technology is concerned, all our research is only for users' reference. The final diagnosis and treatment of diseases still need to rely on doctors.

## Figures and Tables

**Table 1:** Entity and relational data statistics related to hepatitis B.

Data sources	Entities	Relations	Entity types	Relation types
Xunyiwenyao web	3,954	71,247	8	10
Medical records	4,598	25,649	24	29
Counts	8,563	96,896	32	39

**Table 2:** Partial matching templates.

Question contains words	Entities
symptoms; manifestations; phenomenon	Symptoms
prevent; resist; avoid; how we can not	Prevent
diet; drink; eat; taboo; recipe; edible; food; supplement	Food
prescriptions; Chinese patent medicines; Chinese medicine combinations	Prescription

## Data Availability

The medical records of TCM diagnosis and treatment of hepatitis B used in this study are all reviewed and approved by the ethics committee of the Institute of Basic Clinical Medicine of TCM (a subordinate research institute of the Chinese Academy of Chinese Medical Sciences). The approval number of the ethics committee is as follows: 2010 NO 12. We published many papers during the research period [31–33].

## References

[1] Zhou X., Chen S., Liu B. (2010). Development of traditional Chinese medicine clinical data warehouse for medical knowledge discovery and decision support. *Artificial Intelligence in Medicine*.

[2] Zhou X., Peng Y., Liu B. (2010). Text mining for traditional Chinese medical knowledge discovery: a survey. *Journal of Biomedical Informatics*.

[3] WHO (2017). *Global hepatitis report*.

[4] Nayagam S., Chan P., Zhao K. (2021). Investment case for a comprehensive package of interventions against hepatitis B in China: applied modeling to help national strategy planning. *Clinical Infectious Diseases*.

[5] Yunxia W. (2021). Epidemiological analysis and preventive measures of hepatitis B virus in a hospital in Taiyuan City in 2018. *Clinical Medicine Practice*.

[6] Miao Q., Mingzhu G., Yingmei Z., Weizhi C., Min S., Xisheng S. (2020). Research on the mechanism of Yinchenhao decoction in the treatment of viral hepatitis B based on network pharmacology. *Journal of Liaoning University of Traditional Chinese Medicine*.

[7] Cui W., Xiao Y., Wang H., Song Y., Hwang S. W., Wang W. (2017). KBQA. *Proceedings of the VLDB Endowment*.

[8] Li M., Lu X., Chen L., Wang J. (2020). Knowledge map construction for question and answer archives. *Expert Systems with Applications*.

[9] Singhal A. (2012). *Introducing the knowledge graph: things, not strings*.

[10] Fensel D., Şimşek U., Angele K. (2020). *Introduction: What Is a Knowledge Graph?[M]//Knowledge Graphs*.

[11] Mustapha H. (2015). Solution of P versus NP problem. *Algorithms Research*.

[12] Yang Z., Wang Y., Gan J., Li H., Lei N. (2021). Design and research of intelligent question-answering(Q&A) system based on high school course knowledge graph. *Mobile Networks and Applications*.

[13] Cao Y., Liu F., Simpson P. (2011). AskHERMES: an online question answering system for complex clinical questions. *Journal of Biomedical Informatics*.

[14] Sharma S., Patanwala H., Shah M., Deulkar K. (2015). A survey of medical question answering systems. *International Journal of Engineering and Technical Research (IJETR) ISSN*.

[15] Cairns B. L., Nielsen R. D., Masanz J. J. The MiPACQ clinical question answering system.

[16] Mingyu C., Qingqing L., Zhihao Y. (2019). A question answering system for primary liver cancer based on knowledge graph. *Journal of Chinese Information Processing*.

[17] Ma C. H. (2018). Design and implementation of automatic question answering system based on thyroid knowledge map. *Intelligent computers and applications*.

[18] (2016). *Chinese medicine diagnostics*.

[19] Naili Y. (2000). *Differential Diagnosis of Traditional Chinese Medicine Symptoms*.

[20] (2015). *Pharmacopoeia of the People’s Republic of China. I.*.

[21] Consensus Working Group on Chronic Hepatitis B Treatment of Asia-Pacific Society of Hepatology (2012). Cons-ensus on treatment of chronic hepatitis B in the Asia-Pacific region (2012 latest edition). *Journal of C-linical Hepatobiliary Diseases*.

[22] (2019). Guidelines for Diagnosis and Treatment of Chronic Hepatitis B in Traditional Chinese Medicine (2018 Edition). *Journal of Integrated Traditional Chinese and Western Medicine on Liver Diseases*.

[23] Liu Z. (2021). *The Extraction of Clinical Manifestations and Clinical Events from Outpatient Electrical Medical Records of Traditional Chinese Medicine*.

[24] Jixiang Z., Xiangsen Z., Wu C., Zengshun Z. (2021). *Summary of knowledge graph construction technology [J/OL]*.

[25] *The Neo4j Cypher Manual v4.1- Chapter 1. Introduction. [2020-07-03]*.

[26] Huang Z., Xu W., Yu K. Bidirectional LSTM-CRF models for sequence tagging. http://arxiv.org/abs/1508.01991.

[27] Li F., Zhang M., Tian B., Chen B., Fu G., Ji D. (2018). Recognizing irregular entities in biomedical text via deep neural networks. *Pattern Recognition Letters*.

[28] Unger C., Lehmann J., Ngomo A. C. N., Gerber D., Cimiano P. Template-based question answering over RDF data.

[29] Lukovnikov D., Fischer A., Lehmann J. Neural network-based question answering over knowledge graphs on word and character level. *International World Wide Web Conferences Steering Committee*.

[30] Mikolov T., Sutskever I., Chen K., Corrado G. S., Dean J. (2013). Distributed representations of words and phrases and their compositionality. *Advances in Neural Information Processing System*.

[31] Lei Z., Zhou Xuezhong Y., Jian W. Y., Qiming Z. (2013). Study on medication rule of 592 effective cases of chronic hepatitis B with damp-heat syndrome. *Journal of Traditional Chinese Medicine*.

[32] Lei Z., Wang Yiguo Y., Jian Z. X., Zhen L., Wei Z., Qiming Z. (2012). Research on the replacement of prescription drugs in past generations based on clinical data of major infectious diseases. *Chinese Journal of Basic Medicine in Traditional Chinese Medicine*.

[33] Xingwen Y., Lei Z., Yidi C., Qingyue G. (2019). Research on prescription dosage compatibility of traditi-onal Chinese medicine based on fuzzy clustering and fuzzy association. *Chinese Journal of Traditional Chinese Medicine*.

